# Metabolic Profiling Analysis of the Alleviation Effect of Treatment with Baicalin on Cinnabar Induced Toxicity in Rats Urine and Serum

**DOI:** 10.3389/fphar.2017.00271

**Published:** 2017-05-17

**Authors:** Guangyue Su, Gang Chen, Xiao An, Haifeng Wang, Yue-Hu Pei

**Affiliations:** ^1^Department of Traditional Chinese Materia Medica, Shenyang Pharmaceutical UniversityShenyang, China; ^2^Key Laboratory of Structure-Based Drug Design and Discovery, Ministry of Education, Shenyang Pharmaceutical UniversityShenyang, China

**Keywords:** metabolomics, ^1^H NMR, Baicalin, liver and kidney toxicity

## Abstract

**Objectives:** Baicalin is the main bioactive flavonoid constituent isolated from *Scutellaria baicalensis Georgi*. The mechanisms of protection of liver remain unclear. In this study, ^1^H NMR-based metabonomics approach has been used to investigate the alleviation effect of Baicalin.

**Method:** ^1^H NMR metabolomics analyses of urine and serum from rats, was performed to illuminate the alleviation effect of Baicalin on mineral medicine (cinnabar)-induced liver and kidney toxicity.

**Results:** The metabolic profiles of groups receiving Baicalin at a dose of 80 mg/kg were remarkably different from cinnabar, and meanwhile, the level of endogenous metabolites returned to normal compared to group cinnabar. PLS-DA scores plots demonstrated that the variation tendency of control and Baicalein are apart from Cinnabar. The metabolic profiles of group Baicalein were similar to those of group control. Statistics results were confirmed by the histopathological examination and biochemical assay.

**Conclusion:** Baicalin have the alleviation effect to the liver and kidney damage induced by cinnabar. The Baicalin could regulate endogenous metabolites associated with the energy metabolism, choline metabolism, amino acid metabolism, and gut flora.

## Introduction

The toxicity of mineral drugs has been a serious problem that has received increasing attention, especially commonly used heavy metal-containing medicine, cinnabar. Cinnabar (96% as HgS), a commonly used mineral in TCM (traditional Chinese medicine), has been used as sedative agent for a long time (Liu et al., 2008). Mercury is a toxic heavy metal, and mercury could produce toxicity effects on the kidneys, liver, brain and other organs. However, cinnabar has such strong pharmacological effects (Wang et al., 2007) that many TCM prescriptions contain cinnabar. According to the statistics, the number of cinnabar-containing TCM is 46, and the total dose of cinnabar in these prescriptions is dozens of times higher than the allowable dose of 0.1–0.5 g in the Pharmacopeia of China in 2010 (Zhou et al., 2009). Previous studies have indicated that cinnabar could induce strong liver and kidney toxicity at a dose of 1.8 g/kg (Wang et al., 2013). Therefore, it is necessary to identify the detoxification ingredients that alleviate the liver and kidney toxicity caused by cinnabar.

Baicalin is the main bioactive flavonoid constituent isolated from *Scutellaria baicalensis Georgi*. Modern pharmacological research has demonstrated baicalin possesses many biological activities, such as anti-atherosclerotic (He et al., 2016) and anti-fibrotic effects (Peng et al., 2009), cell apoptosis inhibition in breast cancer (Chung et al., 2015), and anticancer activity (Yu et al., 2013). Baicalin reduces the acute hepatic injury induced by CCl_4_ and promotes early recovery in liver function (Taira et al., 2004). Baicalin inhibits hepatocellular carcinoma (HepG2 cells) through oxidative/nitrative stress (Xu et al., 2012). Moreover, baicalin can prevent idiopathic pulmonary fibrosis-induced lipid disorders of the liver (Hu et al., 2015). Additionally, baicalin reduces iron overload-induced rat liver injury by alleviating hepatic pathological damage and decreasing serum alanine aminotransferase (ALT) and Aspartate aminotransferase (AST) activities (Zhang et al., 2012). Therefore, as the above reports suggest, baicalin has a liver protective effect, but these mechanisms of protection remain unclear.

Metabolomics is a rapidly emerging area of “-omics” research that is defined as the qualitative and quantitative analysis of all low molecular weight metabolites. Metabonomics is considered an important platform of systems biology (Feng et al., 2014), and it has been applied for elucidating the drug toxicity mechanisms by taking advantage of the analytical power of modern NMR (nuclear magnetic resonance) and MS (mass spectrometry) instruments (Lindon et al., 2004; Lenz et al., 2005). It plays an important role in investigating the biochemical pathways and potential biomarkers involved in the biological processes (Su et al., 2016).

The pharmacological effects of baicalin were recently demonstrated. However, the liver and kidney improvements following the administration of baicalin in rats with cinnabar-induced acute hepatotoxicity and nephrotoxicity have not been studied. Additionally, the mechanism for this protection, especially the changes in endogenous metabolites as part of metabolic profiling with baicalin, remain unknown. Therefore, in the present study, ^1^H NMR-based metabonomic approach was applied to investigate the liver and kidney alleviation effect of baicalin treatment through comparing the changes in the metabolic profiles of urine and serum between rats treated with baicalin and cinnabar as well as to identify a potential protection mechanism of baicalin treatment.

## Materials and Methods

### Sample and Reagents

**FIGURE 1:**
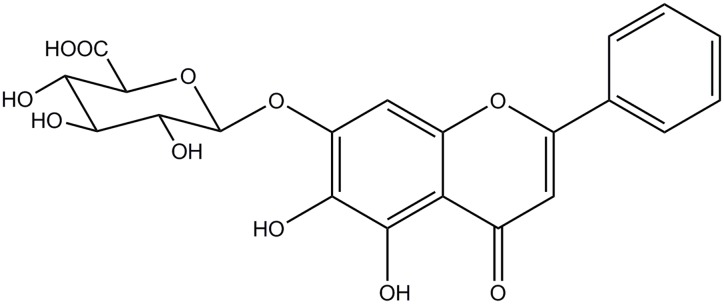
**The structure of Baicalin**.

Baicalin (BE) was purchased from Sigma-Aldrich (St. Louis, MO, United States) And the structure of Baicalin is shown in **Figure 1**. 2, 2, 3, 3-Deuterotrimethylsilylpropionic acid (TSP) was purchased from Norell, Inc. Phosphate buffer was prepared by mixing0.2 M Na_2_HPO_4_ and 0.2 M NaH_2_PO_4_ (pH 7.4 Sigma, St. Louis, MO, United States). D_2_O was purchased from NORELL. Inc., United States.

### Animals and Drug Administration

Twenty-four male Wistar rats (SPF, specific pathogen free) weighing 200 ± 20g (Animal license No. was SYXK-2014-0004) were purchased from Shenyang Pharmaceutical University Experimental Animal Center (China). All work in this study was carried out in accordance with national legislations of China and local guidelines. Animal care and use were approved by the Medical Ethics Committee of the Shenyang Pharmaceutical University (Permit Number: 2014-228). The rats were maintained individually in metabolism cages with a 12 h: 12 h light: dark cycle after acclimatization for 7 days in plastic cages. Food and tap water were provided *ad libitum*. The rats were randomly divided into four groups with six rats in each group that received intragastrically (i.g.) injected water, co-administration of Baicalin and Cinnabar dose group (Group HBC, 80 mg/kg), co-administration of Baicalin and Cinnabar low-dose group (Group LBC, 40 mg/kg) and cinnabar alone (Sun et al., 2010). Cinnabar was injected at doses of 1.8 g/kg (Wang et al., 2013).

### Sample Collection

Urine samples were collected over dry ice in tubes containing sodium azide (1ml 1%) overnight (from PM 10:00 to AM 8:00) on pre-dose day -1 and on post-dose days 1, 2, 3, 5, 6, 7, and 8. Then, they were immediately frozen and stored at -20°C until urine analysis.

On day 8, all animals were sacrificed and blood was drawn from the inferior vena cava. Serum was obtained by centrifugation at 14,000 rpm for 10 min at 4°C. All serum samples were kept at -80°C until further analysis.

### Serum Biochemical Analysis and Histopathology Examination

The clinical biochemical assays of serum samples were per- formed by an automated Hitachi Analyzer (Hitachi Medical Corporation, Tokyo, Japan), including the following parameters: AST, ALT, alkaline phosphatase (ALP), glucose (GLU), creatinine (CREA), triglyceride (TG), total protein (TP), and carbamide (UREA).

A sample of the liver and kidney tissues was immersed in 10% neutral buffered formaldehyde solution; afterward, the tissues were dehydrated, embedded in paraffin, cut at 5 μm thickness. The sliced sections were stained with hematoxylin and eosin (H&E), and examined by light microscopy (200×). Blood for biochemistry measurements was collected and biochemical examination was performed. Data are presented as the mean ± standard deviation (SD).

### ^1^H NMR Spectroscopic Measurement of Urine and Serum Samples

Urine samples (400 μl) were mixed with 200 μl of phosphate buffer (0.2 M, pH 7.4) and centrifuged at 3500 rpm for 10 min to remove insoluble impurities. Supernatants (450 μl) were placed into 5-mm NMR tubes containing 50 μl of TSP (0.1%, w/v) and 60 μl D_2_O.

The serum samples were thawed and 350 μl of serum was mixed with 60 μl of D_2_O and 50 μl of TSP. The samples were then transferred to 5 mm NMR tubes. TSP acted as an internal standard reference (δ 0.00 ppm).

### ^1^H NMR Spectral Data Reduction and Pattern Recognition

All urine and serum spectra were manually phase and baseline adjusted. They were then automatically integrated with MestreNova (8.0.1) software. The chemical shifts of spectra were referenced to the TSP at δ 0.00. The integrals of these buckets covered the region of δ 0.0–9.2 and they were input as variables for principal component analysis-discriminant analysis (PLS-DA). The region of δ 4.6–6.2 was excluded to eliminate the effect of water suppression and urea signals. The data were transferred into an.xsl format (Microsoft Excel 2007, Microsoft, and Redmond, WA, United States) and the integrated data were normalized to the total integrals of each spectrum for partial least squares-discriminant analysis (PLS-DA).

All ^1^H NMR spectra underwent PLS-DA using the software Simca-P^+^ 13.0 (Umetrics, Sweden). Data were visualized by plotting scores and loadings obtained from Partial least squares-discriminant analysis (PLS-DA). The parameters (R2X, R2Y, and Q2) determining the goodness of fit and prediction were determined by SIMCA-P for internal validation. Additionally, permutation tests should be presented for external validation to evaluate the fitting of the model visually. The VIP (variable importance) value, generated in PLS-DA processing, represents the contribution to the group discrimination of each metabolite.

### Statistical Analysis

Statistical analyses of clinical biochemistry data were performed using two-way analysis of variance (2-way ANOVA) followed by the *post hoc* Dunnett’s test. A *p*-value less than 0.05 was considered statistically significant. All data were expressed as the mean ± SD.

### Pathway Analysis

Seventeen biomarkers were subjected to pathway analysis based on metaboanalyst 3.0^1^, and the associated metabolic pathways of each substance with their FDR values are summarized.

## Results and Discussion

### Clinical Biochemistry and Histopathology

To verify the hepatoprotective effects of Baicalin, the AST, ALT, ALP, TP, UREA, CREA, TG, CHO, and GLU serum levels were measured. Biochemical changes of the serum samples are presented in **Table 1**. Significantly increased serum AST, TP, and ALP levels were found in the Cinnabar group compared with the control group. It is worth noting that when treated with Baicalin + Cinnabar (80 mg/kg), the AST, TP, and ALP serum levels were close to those in the control group. There are no significant changes in the UREA, GLU and CREA levels among the four groups.

**Table 1:** Summary of serum clinical biochemistry parameters.

Biochemical parameters	Control	Cinnabar group	Baicalin+ cinnabar (80 mg/kg) group	Baicalin+ cinnabar (40 mg/kg) group
AST (U/L)	92.50 ± 13.07	115.17 ± 16.24^∗^	106.50 ± 16.22	80.60 ± 13.97#
ALT (U/L)	32.08 ± 9.01	39.00 ± 8.72	32.40 ± 5.64	41.67 ± 2.80
ALP (U/L)	193.83 ± 22.27	262.17 ± 37.82^∗^	205.80 ± 74.34	215.17 ± 26.39
TP (g/L)	58.58 ± 11.94	75.62 ± 6.74^∗^	63.56 ± 11.43	67.35 ± 7.78
UREA (lmol/L)	12.88 ± 3.70	10.87 ± 0.86	10.74 ± 1.67	12.05 ± 2.05
CREA (lmol/L)	21.34 ± 4.66	18.17 ± 3.23	17.00 ± 2.83	20.83 ± 5.04
TG (mmol/L)	0.64 ± 0.21	0.72 ± 0.22	0.62 ± 0.30	0.62 ± 0.20
CHO (mmol/L)	1.65 ± 0.46	1.52 ± 0.34	1.51 ± 0.34	1.68 ± 0.15
GLU (mmol/L)	13.78 ± 2.45	14.53 ± 1.49	13.97 ± 2.22	15.79 ± 1.57^∗^


*Data are presented as mean ± SD of six animals per group. ^∗^*p* < 0.05 when compared to control. ^#^*p* < 0.05 when compared to cinnabar.*

**FIGURE 2:**
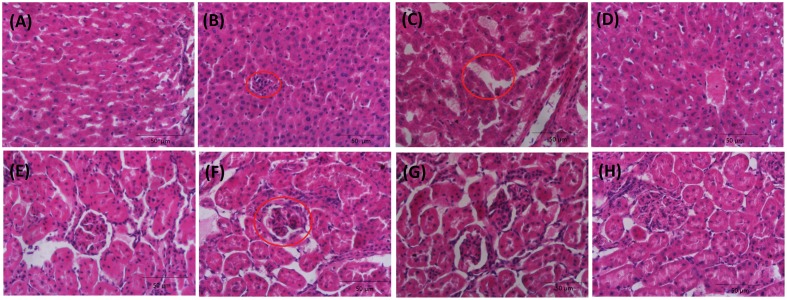
**Kidney and liver histopathology of rats: Liver: control** **(A)**; cinnabar **(B)**; LBC group **(C)**; HBC group **(D)**; Kidney: control **(E)**; cinnabar **(F)**; LBC group **(G)**; HBC group **(H)**. Inside the red circle, **(B)** spotty necrosis. **(C)** Hepatic sinusoidal edema **(F)** glomerular atrophy, lobulated.

Microscopy was used to evaluate the liver (**Figures 2A–D**) and kidney (**Figures 2E–H**) sections from four groups of rats. There are no signs of apparent abnormalities in the livers and kidneys from the control animals. Slight necrosis, swelling of hepatocytes and glomerular atrophy, lobulated are observed in rats treated with cinnabar. Rats in the LBC group showed marked recovery for liver and kidney injuries induced by cinnabar. To conclude, baicalin has good detoxification function of liver and kidney toxicity caused by cinnabar.

### Analysis of ^1^H NMR Spectral Data for Urine

**FIGURE 3:**
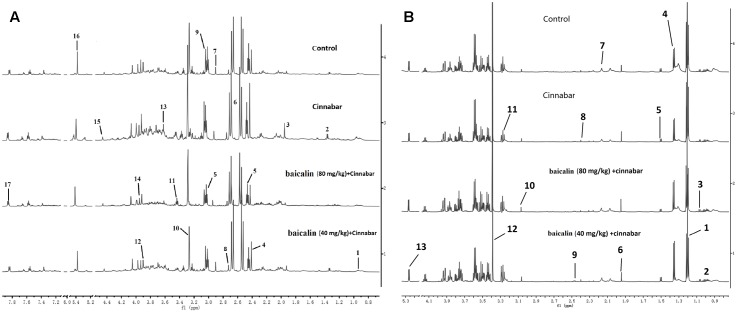
**(A)** 600 MHz spectra of urine obtained from rats from the Control, cinnabar, HBC group and LBC group. Key: 1, leucine+isoleucine; 2, lactate; 3, acetate; 4, pyruvate; 5, 2-ketoglutarate; 6, citrate; 7, dimethylglycine; 8, dimethylamine; 9, creatine; 10, trimethylamine-N-oxide; 11, taurine; 12, betaine; 13, glycine; 14, creatinine; 15, malate; 16, allantoin; 17, hippurate. 2 **(B)** 6 00MHz spectra of serum obtained from rats from the Control, cinnabar, HBC group and LBC group. Key: 1, VLDL/LDL-CH_2_-; 2, leucine+isoleucine; 3, valline; 4, lactate; 5, alanine; 6, acetate citrate; 7, pyruvate; 8, 2-ketoglutarate; 9, citrate; 10, creatine; 11, choline; 12, trimethylamine-N-oxide; 13, unsaturated lipid.

**FIGURE 4:**
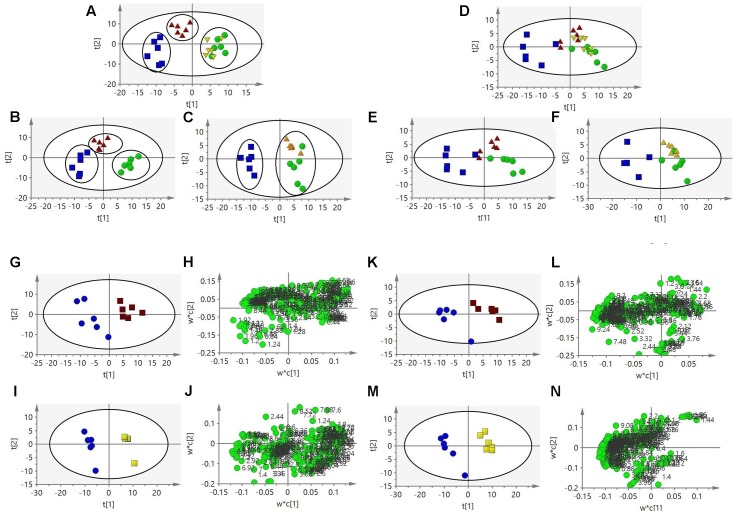
**Partial least squares-discriminant analysis scores plot** **(A)** based on ^1^H NMR spectra of urine from the four groups. PLS-DA scores plot **(B,C)** from the three groups. PLS-DA scores plot **(D)** based on ^1^H NMR spectra of serum from the four groups. PLS-DA scores plot **(E,F)** from the three groups. PLS-DA scores plot **(G)** and corresponding loadings plot **(H)** based on ^1^H NMR spectra of urine from control group and LBC group. PLS-DA scores plot **(I)** and corresponding loadings plot **(J)** based on ^1^H NMR spectra of urine from control group and HBC group. PLS-DA scores plot **(K)** and corresponding loadings plot **(L)** based on ^1^H NMR spectra of serum from control group and LBC group. PLS-DA scores plot **(M)** and corresponding loadings plot **(N)** based on ^1^H NMR spectra of serum from control group and HBC group. Key: cinnabar group (Blue), control group (Green), LBC group (Red), HBC group (Yellow).

A number of endogenous metabolites were observed in the ^1^H NMR spectra of urine from the control, Cinnabar, HBC and LBC groups on day 8 (**Figure 3A**). Assignments of endogenous metabolites involved in ^1^H NMR spectra were based on the literature (Jung et al., 2011; Tang et al., 2012). The urinary NMR spectra were dominated by trimethylamine oxide (TMAO), taurine, betaine, glucose, creatine, creatinine, and tricarboxylic acid cycle (TCA cycle) intermediates, including α-ketoglutaric acid (α-KG), citrate, and succinate. Additionally, a wide range of endogenous metabolites of dimethylamine (DMA), trimethylamine (TMA), dimethylglycine (DMG), hippurate and amino acids, such as leucine, valine, lactate, glycine and phenylalanine, were also apparent (**Figure 3A**). The PLS-DA scores plot based on ^1^H NMR spectra of urine from the four groups are shown in **Figure 4A**.

Partial least squares-discriminant analysis was performed on the urine samples of the control, cinnabar, high-dose Baicalin and Cinnabar (HBC Group; doses of 80 mg/kg), and low-dose Baicalin and Cinnabar (LBC Group; doses of 40 mg/kg) groups on day 8. The HBC and LBC groups are more similar to the control group and less similar to the cinnabar group (**Figures 4B,C**). The PLS-DA score plots reveals that the HBC and LBC groups could be easily distinguished from the cinnabar group along t1 (**Figures 4G,I**). The established PLS-DA model with cross-validation could describe 75.5% of the variation in X (R2X = 0.75) and 75.5% of the variation in the response Y (class; R2Y = 0.68) with a predictive ability of 60.8% (Q2Y = 0.608). The corresponding loading plot (**Figures 4H,J**) showed that, compared with the control and HBC groups, increased alanine, lactate, TMAO, taurine, betaine, hippurate, phenylalanine and glucose; decreased α-OG, succinate, citrate, glycine, creatine and creatinine; and a reduction in the intensities of 2-oxoglutarate, citrate, TMAO and hippurate were observed in the cinnabar group. The ^1^H NMR- relative integral levels of metabolites in urine samples from the different groups are shown in **Table 2**.

**Table 2:** ^1^H NMR-relative integral levels of metabolites in urine samples of groups control, cinnabar, HBC, LBC.

Metabolites	Chemical shift (ppm)	Control group	Cinnabar group	HBC group	LBC group
Lactate	1.32 (d), 4.14 (q)	1.23 ± 0.31	1.98 ± 0.36^∗^	1.24 ± 0.22	1.31 ± 0.24
Alanine	1.48 (d)	1.09 ± 0.09	1.13 ± 0.15	1.11 ± 0.12	1.07 ± 0.17
Acetate	1.93 (s)	1.45 ± 0.22	1.68 ± 0.31	1.51 ± 0.17	1.64 ± 0.23
Succinate	2.41 (s)	2.88 ± 0.25	0.76 ± 0.28^∗^	2.69 ± 0.34	2.52 ± 0.35
α-Oxoglutarate	2.47 (t), 3.01 (t)	3.54 ± 0.26	2.09 ± 0.35^∗^	3.34 ± 0.38	3.29 ± 0.31
Citrate	2.54 (d), 2.66 (d)	5.15 ± 0.47	3.26 ± 0.31^∗^	5.12 ± 0.21	4.97 ± 0.29
Creatine	3.04 (s)	0.98 ± 0.08	2.16 ± 0.15^∗^	1.04 ± 0.11	1.12 ± 0.12
Choline	3.20 (s)	0.29 ± 0.05	0.95 ± 0.09^∗^	0.36 ± 0.08	0.33 ± 0.07
Taurine	3.25 (t), 3.42 (t)	0.26 ± 0.05	0.64 ± 0.08^∗^	0.31 ± 0.04	0.33 ± 0.09
TMAO	3.27 (s)	4.23 ± 0.35	2.05 ± 0.53^∗^	4.17 ± 0.25	4.02 ± 0.28
Betaine	3.89 (s)	1.59 ± 0.21	2.88 ± 0.26^∗^	1.45 ± 0.13	1.42 ± 0.22
Creatinine	4.06 (s)	1.16 ± 0.12	1.23 ± 0.15	1.18 ± 0.09	1.21 ± 0.14
Hippurate	7.55 (t), 7.64 (t), 7.84 (d)	1.35 ± 0.22	0.58 ± 0.18^∗^	1.28 ± 0.15	1.25 ± 0.13


*Multiplicity: s, single; d, double; t, triplet; q, quartet; m, multiplet. ^∗^*p* < 0.05, when compared to control.*

Succinate, citrate and α-OG are vital intermediates in the TCA cycle, and they were all decreased in the cinnabar group, which may be from down regulation of the TCA cycle. The TCA cycle is the core metabolic pathway for energy, which promotes the oxidative decarboxylation of acetyl-CoA and produces reduced equivalents, FADH2 and NADH (Martínez-Reyes et al., 2016). However, succinate, citrate and α-OG have a tendency of reversion in the HBC and LBC groups, indicating that Baicalin may improve hepatic metabolic function through adjusting the endogenous metabolites associated with energy metabolism.

It is important to note that peroxisomes and mitochondria play key roles in various hepatic metabolic functions, including lipid metabolism and energy production, and dysfunction in these organelles is linked to various disorders that affect the liver (van Zutphen et al., 2016). In agreement with the results, we observed high urinary alanine and lactate levels in the cinnabar group; alanine and lactate are intermediates of anaerobic metabolism. Increases in urine amino acids, such as alanine and phenylalanine, were observed in the rats that were treated with Cinnabar, signaling disruption of hepatic amino acid metabolism. However, Baicalin prevented an abnormal increase in the alanine and lactate levels induced by cinnabar.

Trimethylamine oxide and betaine, which could protect cells from damage (Zerbst-Boroffka et al., 2005), were elevated in the Cinnabar Group rats. The formation of hydropic degeneration in response to the hypotonic conditions of the extracellular fluid could correlate with the higher TMAO and betaine levels. TMAO is a product of choline degradation. Decreased TMAO levels in the urine are likely related to disruption of the intestinal bacteria (Xu et al., 2013). The results show the Baicalin could recover the abnormal decrease in TMAO levels induced by cinnabar, which illustrates that Baicalin might regulate the intestinal bacteria.

Additionally, the urinary excretion of creatine and creatinine were significantly reduced, indicating impaired glomerular filtration function induced by Cinnabar. Obvious kidney injury and dysfunction was demonstrated by the histopathologically observed vascular dilatation and congestion in the nephrons as well as by the elevated plasma CREA noted in the biochemical analysis. All changes in the concentrations of the biomarkers and histopathology results suggested that Baicalin has alleviation effect against the liver and kidney damage induced by cinnabar.

### Analysis of ^1^H NMR Serum Spectral Data of Serum

^1^H NMR measurements of the serum demonstrated spectra containing signals from low molecular weight metabolites, and representative spectra of the serum samples are presented. The metabolite signals that were assigned in the proton region of the ^1^H NMR spectra include signals for creatinine, creatine, valine, alanine, TMAO, pyruvate, choline, and lactate (**Figure 3B**).

The list of the assigned metabolites as well as their relative variations (according to PLS-DA) is given in **Table 3**. The PLS-DA scores plot (**Figures 4D–F**) shows that samples in the Cinnabar groups were discriminated well from the control, HBC and LBC groups. A remarkable increase in the alanine and lactate levels was observed. The loadings plot shows that the separation is attributed to the elevation in the of creatinine, acetate, pyruvate, creatine and choline levels. The ^1^H NMR-detected relative integral levels of metabolites in the serum samples of different groups are shown in **Table 3**.

**Table 3:** ^1^H NMR-relative integral levels of metabolites in urine samples of groups control, cinnabar, HBC, LBC.

Metabolites	Chemical shift (ppm)	Control group	Cinnabar group	HBC group	LBC group
Leucine	0.94 (d)	0.62 ± 0.08	1.08 ± 0.08^∗^	0.56 ± 0.05	0.65 ± 0.03
Isoleucine	0.99 (t), 1.02 (d)	0.58 ± 0.07	1.22 ± 0.09^∗^	0.64 ± 0.06	0.61 ± 0.04
valine	1.00 (d), 1,06 (d)	1.02 ± 0.08	1.65 ± 0.12^∗^	1.12 ± 0.15	1.15 ± 0.17
alanine	1.50 (d)	1.54 ± 0.12	0.43 ± 0.08^∗^	1.37 ± 0.15	1.25 ± 0.18
lactate	1.32 (d), 4.14 (q)	4.63 ± 0.51	9.78 ± 1.03^∗^	5.84 ± 0.91	6.36 ± 0.72
α-OG	2.47 (m)	1.56 ± 0.12	0.68 ± 0.14^∗^	1.49 ± 0.13	1.37 ± 0.23
choline	3.20 (s)	3.55 ± 0.18	6.36 ± 0.52^∗^	3.66 ± 0.21	3.87 ± 0.39
creatine	3.07 (s)	0.32 ± 0.05	0.89 ± 0.04^∗^	0.38 ± 0.03	0.36 ± 0.03
pyruvate	2.41 (s)	0.28 ± 0.04	0.67 ± 0.06^∗^	0.35 ± 0.08	0.38 ± 0.07
TMAO	3.27 (s)	2.75 ± 0.28	0.92 ± 0.19^∗^	2.52 ± 0.22	2.45 ± 0.35


*Multiplicity: s, single; d, double; t, triplet; q, quartet; m, multiplet. ^∗^*p* < 0.05, when compared to control.*

The PLS-DA analysis shows that the serum samples from the HBC and LBC groups are distinctly separated from the cinnabar group (**Figures 4K,M**). Additionally, the loadings plot (**Figures 4L,N**) shows that the separation is attributed to the decreased creatinine, creatine, valine, alanine, TMAO, with increased choline, pyruvate and lactate levels.

Decreases in the numbers of branched-chain amino acids (leucine and valine) in serum were observed in the cinnabar separated. A recovery trend of alanine in serum was also observed after combination treatment with Baicalin and cinnabar. This implies that Baicalin helps maintain a balance of hepatic amino acid metabolism. Additionally, loss of the absorption capacity of the proximal tubules by cinnabar increases the excretion of amino acids like alanine, isoleucine, leucine and valine. Meanwhile, we found Baicalin could overturn the reduction tendency in alanine, phenylalanine and lactate, indicating that Baicalin could prevent the liver and kidney damage induced by cinnabar.

Taurine possesses many vital properties, such as anti-oxidation effects, Ca^2+^ flux regulation, membrane stabilization, osmoregulation, and attenuation of apoptosis (Rashid et al., 2013). Increased taurine has long been identified as a specific marker of liver toxicity in serum (Waterfield et al., 1991). In this study, taurine was elevated in the Cinnabar group, which was accompanied by necrosis and steatosis and correlated with the hepatocyte necrosis observed by histopathology. Furthermore, the significant increase in the serum AST levels also supported the presence of hepatic injury. However, taurine was limited in the combined Baicalin and cinnabar (80 mg/kg) group. The taurine level was close to the control group in the Baicalin (80 mg/kg) group. Baicalin effectively protected against liver toxicity.

### Pathway Analysis

**FIGURE 5:**
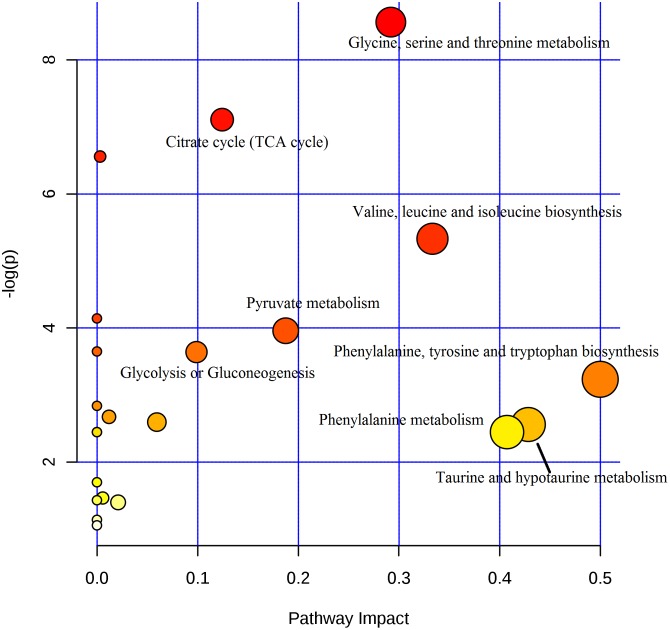
**The summary of pathway analysis.** The *X* axis represents pathway impact, and the *Y* axis represents the -log (p).

**FIGURE 6:**
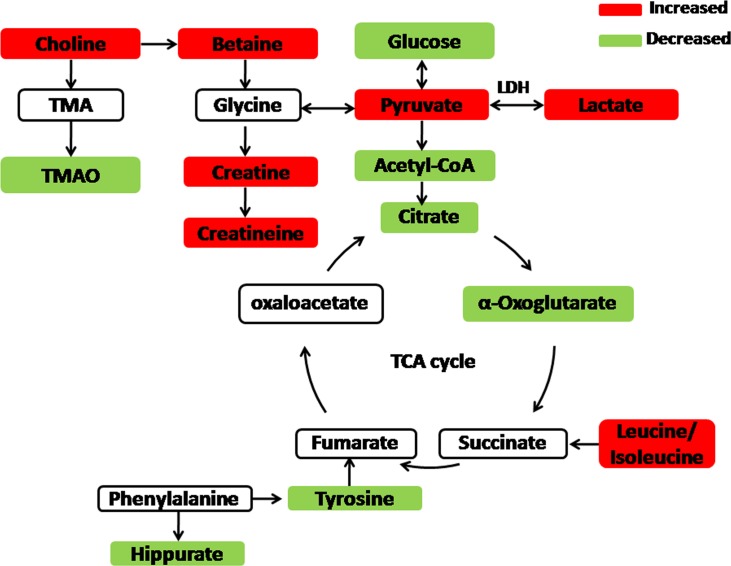
**The schematic representation of the metabolic network**.

Seventeen biomarkers were subjected to pathway analysis based on metaboanalyst 3.0^2^, and the associated metabolic pathways of each substance with their FDR values are summarized in **Figure 5** and Supplementary Table S1. In this study, the taurine and hypotaurine metabolism; phenylalanine metabolism; valine, leucine and isoleucine biosynthesis; pyruvate metabolism; citrate cycle (TCA cycle) pathways were important metabolic pathways with impact factors of 0.43, 0.41, 0.33, 0.19, and 0.12, respectively. The pathway analysis indicates that the alleviation effect of Baicalin was connected to alterations in energy metabolism. One example is with pyruvate metabolism, which is at the core of the TCA cycle. The TCA cycle is a common final pathway that connects with numerous other pathways. TCA intermediates, such as 2-oxoglutarate, citrate, and succinate, were suppressed in urinary samples. Furthermore, amino acid metabolism was altered in view of increased valine, alanine and taurine levels. The schematic representation of the metabolic network is shown in **Figure 6**.

In summary, the results clearly show that the metabolic profiles of groups receiving Baicalin at the dose of 80 mg/kg were remarkably similar to the control profiles. The above results suggested that Baicalin has alleviation effect that combat the liver and kidney damage induced by cinnabar. Baicalin could regulate endogenous metabolites that are associated with energy metabolism, choline metabolism, amino acid metabolism and gut flora. These results also indicate that Baicalin possesses hepatoprotective effects, which may provide a systematic approach for studying for the protective effect of liver and kidney toxicity caused by heavy metals.

## Author Contributions

Conceived and designed the experiments: HW and Y-HP; Performed the experiments: GS and XA; Anayzed the data: HW and GC; Wrote the paper: HW, GS, and Y-HP.

## Conflict of Interest Statement

The authors declare that the research was conducted in the absence of any commercial or financial relationships that could be construed as a potential conflict of interest. The reviewer KX and handling Editor declared their shared affiliation, and the handling Editor states that the process nevertheless met the standards of a fair and objective review.

## Footnotes

1 http://www.metaboanalyst.ca

2 http://www.metaboanalyst.ca
