# Supplementary material for: Metabolic Profiling Analysis of the Alleviation Effect of Treatment with Baicalin on Cinnabar Induced Toxicity in Rats Urine and Serum
Source: Front Pharmacol. 2017 May 17;8:271. doi: 10.3389/fphar.2017.00271 (PMC5434134; doi:10.3389/fphar.2017.00271)
Supplement: Supplementary file 1 [file Table_1.docx]

Table S1. The pathway analysis potential target metabolites

|  | compound | Expected | Hits | Raw p | -Log p | Holm adjust | FDR | Impact |
| --- | --- | --- | --- | --- | --- | --- | --- | --- |
| Glycine, serine and threonine metabolism | 32 | 0.31954 | 4 | 0.000191 | 8.5628 | 0.015477 | 0.015477 | 0.29197 |
| Citrate cycle (TCA cycle) | 20 | 0.19971 | 3 | 0.000819 | 7.1078 | 0.065495 | 0.033157 | 0.12438 |
| Alanine, aspartate and glutamate metabolism | 24 | 0.23966 | 3 | 0.001419 | 6.5575 | 0.11214 | 0.038325 | 0.00316 |
| Valine, leucine and isoleucine biosynthesis | 11 | 0.10984 | 2 | 0.00484 | 5.3308 | 0.37754 | 0.098015 | 0.33333 |
| Butanoate metabolism | 20 | 0.19971 | 2 | 0.015882 | 4.1425 | 1 | 0.25729 | 0 |
| Pyruvate metabolism | 22 | 0.21969 | 2 | 0.01909 | 3.9586 | 1 | 0.25772 | 0.18754 |
| Aminoacyl-tRNA biosynthesis | 67 | 0.66904 | 3 | 0.026009 | 3.6493 | 1 | 0.26581 | 0 |
| Glycolysis or Gluconeogenesis | 26 | 0.25963 | 2 | 0.026253 | 3.64 | 1 | 0.26581 | 0.09891 |
| Phenylalanine, tyrosine and tryptophan biosynthesis | 4 | 0.039943 | 1 | 0.03939 | 3.2342 | 1 | 0.35451 | 0.5 |
| Cyanoamino acid metabolism | 6 | 0.059914 | 1 | 0.05854 | 2.838 | 1 | 0.43842 | 0 |
| Arginine and proline metabolism | 44 | 0.43937 | 2 | 0.068977 | 2.674 | 1 | 0.43842 | 0.01198 |
| Primary bile acid biosynthesis | 46 | 0.45934 | 2 | 0.074614 | 2.5954 | 1 | 0.43842 | 0.05952 |
| Taurine and hypotaurine metabolism | 8 | 0.079886 | 1 | 0.077335 | 2.5596 | 1 | 0.43842 | 0.42857 |
| Methane metabolism | 9 | 0.089872 | 1 | 0.086602 | 2.4464 | 1 | 0.43842 | 0 |
| Nitrogen metabolism | 9 | 0.089872 | 1 | 0.086602 | 2.4464 | 1 | 0.43842 | 0 |
| Phenylalanine metabolism | 9 | 0.089872 | 1 | 0.086602 | 2.4464 | 1 | 0.43842 | 0.40741 |
| Propanoate metabolism | 20 | 0.19971 | 1 | 0.18299 | 1.6983 | 1 | 0.87187 | 0 |
| Glutathione metabolism | 26 | 0.25963 | 1 | 0.23149 | 1.4632 | 1 | 1 | 0.00573 |
| Porphyrin and chlorophyll metabolism | 27 | 0.26961 | 1 | 0.23931 | 1.43 | 1 | 1 | 0 |
| Cysteine and methionine metabolism | 28 | 0.2796 | 1 | 0.24705 | 1.3981 | 1 | 1 | 0.02103 |
| Valine, leucine and isoleucine degradation | 38 | 0.37946 | 1 | 0.32058 | 1.1376 | 1 | 1 | 0 |
| Tyrosine metabolism | 42 | 0.4194 | 1 | 0.34808 | 1.0553 | 1 | 1 | 0 |

The statistical p-values were calculated from enrichment analysis; the Compounds is the total number of compounds in the pathway; the Hits is the actually matched number from the user uploaded data; the Raw p is the original p-value calculated from the enrichment analysis; the Holm p is the p-value adjusted by the Holm–Bonferroni method; the FDR p is the p-value adjusted using the false discovery rate; the Impact is the pathway impact value calculated from pathway topology analysis.
